# Supplementary material for: Cirrhotic Cardiomyopathy Following Bile Duct Ligation in Rats—A Matter of Time?
Source: Int J Mol Sci. 2023 May 2;24(9):8147. doi: 10.3390/ijms24098147 (PMC10249007; doi:10.3390/ijms24098147)
Supplement: Supplementary file 1 [file ijms-24-08147-s001.zip › Supplementary Figures Uhlig IJMS/Figure S2 Schematic illustration of the study protocol.pdf]

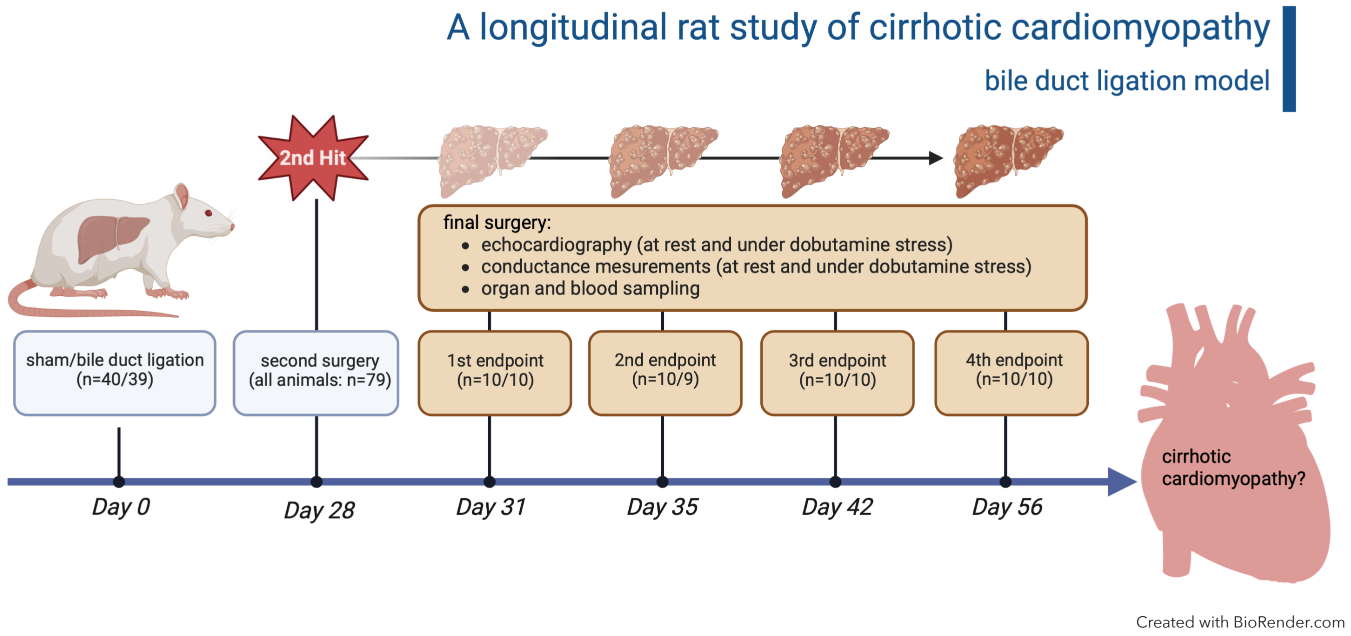


**Figure S2. Schematic illustration of the study protocol**
