# Supplementary material for: Cirrhotic Cardiomyopathy Following Bile Duct Ligation in Rats—A Matter of Time?
Source: Int J Mol Sci. 2023 May 2;24(9):8147. doi: 10.3390/ijms24098147 (PMC10249007; doi:10.3390/ijms24098147)
Supplement: Supplementary file 1 [file ijms-24-08147-s001.zip › Supplementary Figures Uhlig IJMS/Figure S1 Cardiac Remodeling Index.pdf]

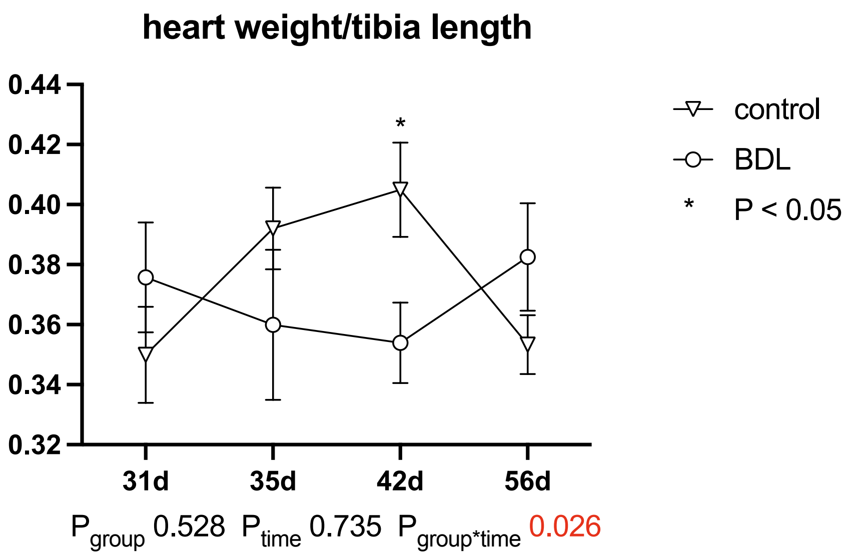

Figure S1. Cardiac remodeling index (CRI) of rat hearts after bile duct ligation (BDL) versus control.
The measured heart weight was normalized to the tibial length of each rat respectively and displayed as mean ratio from day 31 to 56 after BDL (mean ±SEM). $P_{group}<0.528$, $P_{time}<0.735$, $P_{group*time}=0.026$, $^*P_{d42}=0.02$. $P \leq 0.05$ was considered significant.
